# Experiences and perceptions of participants on the pathway towards clinical management of dual tuberculosis and diabetes mellitus in Tanzania

**DOI:** 10.1080/16549716.2022.2143044

**Published:** 2022-11-28

**Authors:** Nyasatu G. Chamba, Kenneth C. Byashalira, Dirk L. Christensen, Kaushik L. Ramaiya, Eliakimu P. Kapyolo, PendoMartha J. Shayo, Troels Lillebaek, Nyanda E. Ntinginya, Blandina T. Mmbaga, Ib C. Bygbjerg, Stellah G. Mpagama, Rachel N. Manongi

**Affiliations:** a Kilimanjaro Christian Medical University College, Faculty of Medicine, Moshi, United Republic of Tanzania; b Department of Internal Medicine, Kilimanjaro Christian Medical Centre, Moshi, United Republic of Tanzania; c Kibong’oto Infectious Disease Hospital, Department of Research, Sanya Juu, United Republic of Tanzania; d Global Health Section, Department of Public Health, University of Copenhagen, Copenhagen, Denmark; e Department of Internal Medicine, Shree Hindu Mandal Hospital, Dar es Salaam, United Republic of Tanzania; f Department of Clinical research, National Institute for Medical Research, Dodoma Medical Research Centre, Dodoma, United Republic of Tanzania; g International Reference Laboratory of Mycobacteriology, Statens Serum Institut, Copenhagen, Denmark; h National Institute for Medical Research, Mbeya Medical Research Centre, Mbeya, United Republic of Tanzania; i Kilimanjaro Clinical Research Institute, Directorate of Research and Consultancies, Moshi, United Republic of Tanzania; j Institute of Public Health, Kilimanjaro Christian Medical University College, Moshi, United Republic of Tanzania

**Keywords:** Tuberculosis, diabetes mellitus, perception, patient centered care, Tanzania

## Abstract

**Background:**

Diabetes mellitus (DM) is a common comorbidity among people with tuberculosis (TB). Despite the availability of guidelines on how to integrate dual TB/DM in Tanzania, the practice of integration at various healthcare levels is unclear.

**Objective:**

To explore the participants’ experiences and perceptions on the pathway towards clinical management of dual TB/DM.

**Method:**

The research was carried out in Dar es Salaam, Iringa, and Kilimanjaro regions between January and February 2020. A qualitative, in-depth interview approach was used to collect participants’ experiences and perspectives on the acquisition of dual TB/DM services at various levels of healthcare facilities. The information gathered were coded and classified thematically.

**Results:**

The participants’ perception of TB services within the healthcare facilities was positive due to the support they received from the healthcare providers. On the other hand, participants reported difficulty receiving management in various health facilities for each condition in terms of access to dual TB/DM care and access to DM medication. This was viewed as a significant challenge for the participants with dual TB/DM.

**Conclusions:**

The current disjunction and disruption in healthcare for people with dual TB/DM makes it difficult to access services at various levels of health facilities. For optimal clinical management for people with dual TB/DM, patient-centered strategies and integrated approaches are urgently needed.

## Introduction

Diabetes mellitus (DM) is a common comorbidity among individuals with tuberculosis (TB), where globally, 10% of TB cases are linked to DM [1]. Over 15% of people with TB worldwide are estimated to have DM. This translates to approximately 1.5 million people with TB and DM who require coordinated care and follow-up to optimize the management of their dual condition [2]. In low- and middle-income countries, the burden of DM is estimated to be 80%, and that of TB to be 90% respectively [3]. A systematic review of 13 observational studies by Jeon et al. confirms that diabetic individuals are at an increased risk of TB, consistent evidence that hyperglycemia affects the host response to TB [4]. The World Health Organization and The International Union Against Tuberculosis and Lung Disease developed the ‘Collaborative framework for care and control of tuberculosis and diabetes’ to address the growing TB/DM co-epidemic and in response to the increasing challenges posed by the dual TB/DM comorbidity, which recommends integrated prevention and care for the dual comorbidity and implementation of TB infection control in healthcare settings where DM is managed [1]. This framework outlines recommendations to guide countries in the care, prevention, and control of TB and DM with a focus on (1) improving detection and management of TB in patients with DM, (2) improving detection and management of DM in patients with TB, and (3) establishing mechanisms of joint coordination within the regional, district, and primary healthcare levels [1].

The integration of dual TB/DM management requires a multidisciplinary team across healthcare settings because DM can aggravate the clinical course of TB, and TB can affect glycemic control in people with DM [5].

Although there are more individuals with TB/DM than TB/HIV co-infection [4,6], an integrated management system for individuals with TB/HIV already exists in Tanzania. A similar integrated approach for TB/DM would emphasize on preventive measures more. A good example would be control measures to prevent airborne *M. tuberculosis* transmission in DM clinics, as studies have shown that current measures are insufficient [7].

Integration and Patient-Centered Care (PCC) services for individuals with TB and comorbidities such as DM are included in Pillar 1 of the End TB strategy [8]. Since 2011, when WHO first recommended collaborative activities to combat TB and DM, the implementation process of PCC for TB and DM has varied. As a result, the WHO introduced a policy to aid in monitoring PCC for TB and DM implementation [8]. In a systematic study, the concept of PCC resulted in positive outcomes primarily in the US and some Canadian hospitals, including decreased malpractice complaints, increased individual satisfaction, reduced consultation time, improved emotional states, and medication adherence [9,10]. Despite the evidence, PCC has proven challenging to implement in many low-income Sub-Saharan African countries’ healthcare systems. Despite all the evidence, implementing PCC in many health care systems within the low-income sub-Saharan African countries has been difficult [11]. In Tanzania, the PCC concept is essential for achieving long-term healthcare coverage within a specific health service, such as dual TB/DM services. Family involvement in healthcare decisions is an important component of PCC, as evidenced by a systematic review that found that family and friend involvement is 60% [12]. Individuals with this dual comorbidity in Tanzania primarily rely on the public health system for TB and DM testing, treatment, and care. Staffing shortages, insufficient equipment, drug stock-outs, insufficient therapy, and poor disease management are all common issues in these public hospitals, jeopardizing PCC delivery [11]. In Tanzania, public health facilities purchase drugs from the Medical Stores Department based on the National Essential Drugs List. Patients receive medications through a cost-sharing scheme in which they must pay half of the drugs’ actual price, including anti-diabetic medications [13] whereas TB drugs are subsidized through Tanzania’s National TB and Leprosy Programme (NTLP).

A typical pathway for accessing service begins with a primary healthcare facility, where a basic workup is performed. The individual is referred to a secondary or tertiary healthcare facility for more complex situations for specialized tests and care [14]. If all of these services are not available in one location, people with dual diseases may need to visit several different levels of health facilities to receive specialized management for each condition. Care pathways are frequently included in integrated care and help guide the delivery of integrated care to patients while clarifying roles and responsibilities in the process [15]. However, due to a lack of clinical integration in Tanzanian’s existing health system, patients’ pathways to obtaining therapy have proven a struggle. We do not have information about dual TB/DM individuals’ treatment plans, challenges, or how they believe these services can be improved for their benefit.

As a result, the purpose of this study was to investigate participants’ experiences and perceptions on the path to dual TB/DM service delivery in healthcare facilities where healthcare professionals were trained with the Adaptive Diseases control Expert Program (ADEPT) in Tanzania [16], guided by the Picker’s Eight Principles of Patient Centered Care [17] as patient-centered strategies.

## Methods

### Study design

This qualitative study used Picker’s Eight Principles of Patient Centered Care, which covered the aspects of: (1) Respect for patients’ values, preferences, and expressed needs; (2) Coordination and integration of care; (3) Information, communication, and education; (4) Physical comfort; (5) Emotional support, and alleviation of fear and anxiety; (6) Involvement of family and friends; (7) Continuity and transition; and (8) Access to care. During the in-depth interview (IDI), the participants’ experiences and perspectives on the pathways in obtaining dual TB/DM services at various levels of healthcare facilities, from diagnosis to treatment initiation of dual TB/DM, were addressed in depth.

### Study setting

Between January to February 2020, study participants were recruited from randomly selected healthcare facilities where healthcare providers, including clinicians and nurses, were trained on the screening and management of dual TB/DM as part of the ADEPT programme in order to implement integrated TB/DM care [16]. There were three regional hospitals, four district hospitals and eight health centers among the health facilities in Dar es Salaam (DSM), Iringa (IRA) and Kilimanjaro (KLM) regions in Tanzania. DSM has the highest burden of DM and is the major contributor of the TB incidence of 129 per 100,000 populations annually [18,19]. The prevalence of HIV in IRA is 11.3% which contributes significantly to the co-infection of TB and HIV in the country [19,20]. The KLM region has a high prevalence of DM and a TB burden of 150 per 100,000 population [19,20]. The participants were all from the out-patient department, and the IDIs were conducted in Swahili, the language spoken by all participants within the hospital premises away from the patients’ clinics and wards to maintain privacy.

### Study participants

All study participants were ≥18 years of age, on TB treatment between the first and sixth months, and those diagnosed with DM, who were taking both anti-TB and hypoglycemic medications, were purposively selected to be in the IDIs. After interviewing the fourteenth participants, we felt we had reached saturation. This was accomplished when no new ideas or information were discovered during the data collection and analysis phase [21]. On average, each interview lasted between 30 to 60 minutes. After explaining the purpose of the study to the participants, they signed a written informed consent for the in-depth interview.

### Data collection

A semi-structured interview guide was used to collect data. The first author (NGC) facilitated the IDI among study participants. The interviewer was able to better understand the participants’ experiences with dual TB/DM services at various levels of healthcare facilities. To better understand the path involved in receiving TB and DM management, open-ended questions were followed by standardized probes. The conversation was audio recorded with the participants’ permission, and the integrated decisions were transcribed verbatim in Swahili, and then translated into English.

### Data analysis

Data were coded to extract relevant information. Transcription and audio recordings were reviewed by NGC and co-authors SGM and RNM for accuracy before conducting content analysis using Hsieh & Shannon standard method [22]. Discrepancies were examined between NGC, SGM and RNM and SGM, and an agreement was reached. The research team (NGC, KCB, DLC, KLR, EPK, PJS, NEN, TL, BTM, ICB, SGM, and RNM), discussed on the main themes and reached a consensus. The themes were classified using Picker’s eight principles of PCC. Data analysis was supported using ATLAS.ti version 9, a qualitative research tool. This paper adhered to the reporting guidelines for qualitative research as outlined in the unified criteria for reporting qualitative studies [23].

## Results

**Figure 1.**
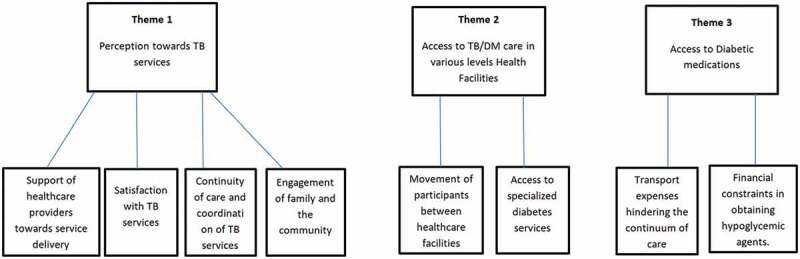
Themes and subthemes.

The findings of this study deductively identified around common themes linked to the Pricker’s eight principles of PCC. As shown in Figure 1, the findings addressed three main themes: the first theme, ‘perception towards TB services’, covered the notion of: respect for patients’ values, preferences, and expressed requirements was explored through the support and satisfaction obtained in terms of continuity and coordination of care, emotional support and alleviation of fear and anxiety, and family and friends involvement. The second theme ‘access to TB/DM care in various levels of health facilities,’ covered the access to care, coordination and integration of care and information. The third theme ‘access to diabetic medications’ covered the notion of continuity and transition of care and access to care.

### Theme 1: perception towards TB services

This theme reflects the participants’ perception of the services they experienced in TB clinics at various levels of health facilities, as participants with dual TB/DM are required to receive TB management within the TB clinics during the intensive phase of treatment.

### Support of healthcare providers towards service delivery

The participants gave positive feedback about their service delivery experience. There was strong agreement among the participants on the good rapport the health care providers established and the services they provided. Their first impression influenced the participants’ ability to express themselves. According to one of the participants, the positive view was primarily based on the services provided in the TB clinic.

When I first came to this TB clinic, the nurses were very welcoming. They received me well. (37-year old female_DSM)

A positive relationship between participants and healthcare providers is important because it influences client satisfaction and trust in the services provided; as a result, it leads to a positive health outcome by increasing individual understanding of his or her health problems and available medications, as outlined below:

To be honest, I think the relationship is good because when I come here to the district hospital for TB medications, the nurses know my name, they welcome me and they are used to me by now. This gives me the freedom to express myself if there are any other issues bothering me. And if there are medications, they prescribe them to me; if not, they tell me those are minor issues with the current medications, and the condition will go away. They are reassuring and give me hope to continue living with these diseases. (40-year old male_KLM)

### Satisfaction with TB services

Participants’ satisfaction encompasses a wide range of interactions with the healthcare system, including the experience in interacting with the healthcare providers, waiting time, counseling, and prescription. The following quotes reflect the satisfaction from the clients, one of them being a competent elderly woman:

When I first arrived at this health center, the nurses told me to wait while they opened a file for me. When I told the doctor about my problem, he advised me to do some investigations. Right now, I am going to the doctor to get TB medications. The good thing is that when I come here, they take care of me quickly so I leave early. (44-year old female_IRA).

I am extremely pleased. I do not have to pay for TB medications to receive them. They (doctors and nurses) talk to me and give me free medical advice. The medications had helped me because I can now do some of the things I used to do before I got sick. (80-year old female_IRA)

### Continuity of care and coordination of TB services

The follow-up clinic visits for TB clients within the healthcare facilities have helped to guarantee continuity of care in terms of drug refills. The return dates and the short messages sent to individuals via their cell phones have helped to remind them to take their TB medications daily.

I come to the clinic on the scheduled dates, and they observe how I use my medications and my progress from when I first started coming until now. I would not have been able to handle myself if I had not used my medications correctly … . They also send me a message every morning at five thirty to remind me to take my medications. To be honest, they are doing an excellent job. They should continue in this manner because I have observed no problem. (59-year old female_DSM)

They attempt to remind me about the medications and the clinic visits. So I cannot miss any of my scheduled dates. They usually give us the return dates and remind us to take our medications on a regular basis. Their communication is very clear, so you will not get confused. If you have a question, they readily answer. (57-year old female_IRA)

The checking of the TB treatment cards, according to the participants, ensured tight supervision in taking the medications thus resulting in dose completion.

When I was first diagnosed with TB, they were extremely strict with me. They told me to swallow the pills in front of them and then return in a week for more medications. They check my TB card to see if I am taking the pills every day, and they tell me to return with the completed package so they can see if I have finished my pills. So I have been going once a week to get my TB medications. (37-year old female_DSM)

Every week when I come to pick up the medications, they have been supervising me taking the TB medications. I always have to bring the TB card with me so that they can inspect it. (50-year old male_KLM)

### Engagement of family and the community

TB is known to be a stigmatizing disease. However, there was a consensus among participants that the involvement of close family members such as parents, children, spouses, and siblings, and the support obtained from neighbors has played a significant role in the continuity of care as reflected in the quotes below:

My family has been extremely supportive. As you know, everyone has their own family, initially; they helped collect the TB medications from the clinic because I do not have a small child to whom I could send. But now that I have regained my energy, I come to the clinic on my own, as I did today. (42-year old male_KLM)

The engagement included escorting the individuals to and from their scheduled TB clinic appointments as well as supporting them in obtaining TB medications.

In many ways, my family has been involved in my treatment such as helping to pay my medical bills when I was admitted in the hospital and ensuring I am eating adequately and appropriately. My wife has been doing an excellent job of reminding me of my clinic days because she accompanies me to the clinics. (69-year old male_KLM)

I only involved my son called Gerald. He is a motorcycle driver, so he assists me in picking up medications on the days when I am feeling ill and am unable to go pick up the medications myself. (57-year old female_IRA)

Adherence to TB treatment is important for controlling the spread of infection and also minimizes the development of drug resistance. To achieve this, the participants’ progress was aided by their assistance from family members and the community.

The doctor told me to bring an escort to support me and supervise my medications. So I informed my children and my niece, who lives in a nearby village to assist me. So when I returned, I mentioned my niece as my next of kin, who will assist me. (80-year old female-Iringa)

I informed my sister, who is my older sister. She has been extremely helpful to me. She always ensures that I take medications on time and do not skip a day. She also rushes back home to cook for me so that she tells me to do so when the time comes for me to take my medications. (44-year old female_IRA)

The community’s involvement, such as that of neighbors, has also aided in the continuity of care for these individuals.

I have asked one of our neighbors in the village to assist me with food while I continue taking the anti-TB medications. She has been very supportive because she has seen how wasted I look because of the disease and how I cannot do much on my own. (42-year old male_KLM)

‘A friend in need is a friend indeed’, as the saying goes, but this was not the case for some individuals. Friends were not present during the illness but only during in the good times.

I did not see any of my friends during my illness. But when I recovered, the house was once again full. (42-year old male_KLM)

To be honest, I have few friends and did not involve them in my illness. You know my friends are busy and I do not want to bother them. (44-year old male_DSM)

I do not need my friends because they loved me when I had money; I have seen none of them since I got sick. Only one of my friends tries to help me when he can, but the others have not, that is why I do not need friends right now. (41-year old male_DSM)

### Theme 2: access to TB/DM care in various levels health facilities

Twelve participants reported receiving treatment for TB and DM in various health facilities. Due to persistent symptoms of uncontrolled DM, confirming the diagnosis and monitoring the progression of the illness, along with refilling DM medications, especially insulin injections, the participants were referred to higher-level health facilities such as district or referral hospitals for DM diagnosis and management.

### Movement of participants between healthcare facilities

The pathway of participants shows that, they had to be referred from a primary healthcare facility to a secondary and even tertiary facility to seek further investigations and management.

I am a visitor in this place. I came here after being referred from my village hospital due to my illness and other investigations. When I arrived, I was greeted by a nurse, who welcomed me and provided services by asking me a few questions before taking me to the doctor where I explained myself to him. (44-year old female_IRA)

I had to go to the Regional hospital because my blood sugar was too high. The main challenge is that I have to go to the health centre for my TB medications and to the regional hospital for my insulin injections. (40-year old male_KLM)

### Access to specialized diabetes services

Integrated management of DM and TB is important to ensure that optimal care is provided to patients. However, the participants experienced movements from one health facility to another seeking for specialized diabetes services which were obtained within the National Hospital in DSM and tertiary hospital in KLM. These movements were reported by the participants.

When I first came to this health center, the nurses were very welcoming and they advised me to go to Muhimbili National hospital for further investigations and when I returned to continue my TB medications, they continued supporting me. (59-year old female_DSM)

I have been receiving diabetes treatment right here at the regional hospital. However, when I was diagnosed with TB, I was told to go to KCMC (Tertiary zonal hospital) to control my blood sugars with medications because I had TB. So I have to come here (regional hospital) for my TB medications and go to KCMC for my diabetes treatment. I go to KCMC once a month for the medications and come here once a week to pick up the TB medications. Given my age, this situation is tedious. (69-year old male_KLM)

### Theme 3: access to diabetic medications

The participants, complained about obtaining the DM medications because they had to travel from one health facility to the next or to pharmacies to obtain them. After all the cost of procuring insulin from pharmacies and the transportation fare moving from one health facility to the next, resulted in inconveniences and poor blood sugar control.

### Transport expenses hindering the continuum of care

Traveling from one health facility to the next has been reported to be expensive and as result, participants do not receive the required medications. The participants disclosed on the hurdles they get when seeking insulin injections.

I use insulin injection for diabetes. So they write a prescription for me to go out and buy. At my age, I cannot afford the costs of travel; I depend on what my children send me. (80-year old female_IRA)

On other days, I do not get injected because I do not have fare, and when I go to the dispensary near my house, they say they do not have syringes. So sometimes I get injected and other times I can go three to four days without being injected. (57-year old female_IRA)

### Financial constraints in obtaining hypoglycemic agents

DM can incapacitate a person if not controlled, therefore a person may not be able to support him/herself or get money to buy the hypoglycemic agents. Due to the financial constraints, the participants reported that they needed money to obtain the diabetes medications; otherwise, they may go days without the hypoglycemic agents.

It is difficult to move from one health facility to the next. I have never forgotten to pick up my TB medications. However, with the diabetes medications, it depends on whether I have money to buy them that day. As a result, there are days I do not take the medications. (69-year old Male_KLM)

I am not sure if I will be able to afford diabetes medications. When they give me the prescription to buy the diabetes medications, I simply go to the hospital pharmacy and tell them that I do not have even a hundred shillings, so they confer and decide to give me at least few pills to swallow for about three days, but on other days, I do not take the diabetes medications. I just hope that when I finish the TB medications I will not have diabetes anymore. (50-year old Male-KLM)

## Discussion

The findings of this study highlighted challenges in the management of dual TB/DM comorbidity, particularly within a health system that is unable to address the integration of dual communicable and non-communicable diseases (NCDs). The participants’ narratives expressed their experiences, impressions, and perceptions of the pathways in obtaining dual TB/DM services at various levels of healthcare facilities. The DM services were concentrated specifically within the regional referral hospitals, as they have a designated DM clinic that operates weekly, and the supply of necessary medications such as insulin was available [24].

The services provided in the TB clinics were satisfactory to the participants. This is because the healthcare providers, particularly the nurses, demonstrated a positive attitude by inspiring, counseling and empathizing with them. Similarly, a study conducted in Ethiopia found that participants’ perceptions of healthcare professional interactions significantly impacted on the individual’s satisfaction and adherence to TB treatment [25]. In our study, the TB clients were properly informed about their illness and the care they received, and they were all satisfied with the education they received and the importance of completing their TB prescriptions. Observer checking, as recommended by the NTLP, was used to ensure TB treatment continuity [19], which is a significant step towards improving the quality of care. According to an exploratory systematic review study, TB individuals were either entirely satisfied or satisfied with the availability and effectiveness of public TB services [26].

Although the basic needs for TB diagnosis and treatment were met, participants’ perspectives revealed various challenges and suggested actions to improve continuity of care for dual TB/DM. These individuals had to travel from one health center, to a district hospital and then to a regional referral hospital to receive dual TB/DM treatment. This process has raised concerns about the transmission of TB as individuals move from the TB clinic to the DM clinic to obtain drugs, either within the same or a different health facility. Despite the presence of infection prevention and control recommendations in the country’s TB health facilities [19], strategies for providing care in one sitting have not been implemented, posing a risk to other clients and employees. A similar observation was reported in South Africa, where infection prevention and control protocols in health facilities were not followed properly [27].

This study found that DM services, in terms of management and follow-up, are available within the referral and district facilities, where individuals were required to move from one health facility to the next in order to obtain DM medications, majority of which were insulin injections due to uncontrolled DM while using TB medications. Healthcare facilities in Tanzania are not sufficiently ready to manage patients with NCDs, including DM, hypertension, and chronic respiratory diseases [28]. This is primarily due to a lack of basic diagnostic equipment, guidelines, and key drugs in most Tanzanian rural health facilities, and insufficient training, administration, and reporting systems for DM and hypertension care [14]. Due to the rising burden of NCDs, the Ministry of Health and Social Welfare has advocated for the decentralization of DM services to lower-level primary care facilities. In order to achieve this, more efforts should be made to provide clinical guidelines, basic diagnostic equipment, medication therapies, and ensure appropriate expertise among frontline health care workers [14]. In low and middle-income countries (LMICs), the decentralization of HIV care and treatment services as well as TB diagnosis and treatment has been done successfully [29]. Similarly, Tanzania’s collaborative TB/HIV activities have spread across the country since 2005, thanks to the development of TB/HIV treatment, operations and standard of care guidelines [29]. According to reports, decentralizing DM services in Ethiopia has improved access and utilization [30].

Because medications are the foundation of DM treatment, they must be easily accessible, affordable, and culturally appropriate for people with DM who will require treatment for the rest of their lives [13]. The cost of DM management affects all individuals with DM. Intangible costs such as discomfort, worry, annoyance, and a lower overall quality of life significantly impact on the lives of people with DM and their families and are the most difficult to quantify [13]. The majority of individuals in our study had difficulty purchasing DM medications because the individuals were not covered by the National Health Insurance Fund. As a result, they were forced to buy the medications. Missed doses and other complications resulted from high cost of obtaining DM medications. Despite being one of the essential medications, insulin is expensive and inaccessible in many LMICs. In many African countries, a vial of insulin can cost as much as a month’s salary, whereas in Tanzania, where cost-sharing mechanisms exist in the public health sector, the cost of one vial of insulin is around US$ 1 [31]. A study conducted in eastern Nigeria found that there are financial barriers to using DM services [32]. To this end, Nolte et al. argue that chronic disease financing systems should reduce out-of-pocket costs to enhance patient’s involvement in their own chronic care [33]. Individuals, families, healthcare systems, and governments are all affected by DM and its consequences [31].

In order to improve access to and quality of DM services in lower-level health facilities, these services must be prioritized. As with HIV, the concept of task shifting has been promoted as a way of rapidly expanding human resources capacity [34] thus ensuring the availability of qualified responsible individuals for DM and other NCDs at the regional, district and facility levels will increase accountability for DM care in rural areas.

### Study limitation and strengths

The study had limitations whereby we could not interview individuals from varying distances to various levels of health services, a strong or a weaker family for informal support and care, varying literacy and health literacy, and assessing individuals with or without access to cell phones. The inclusion of expenditures for blood glucose monitoring, which can sometimes exceed the insulin required to normalize it, would also be beneficial for better TB/DM care integration. It is critical to follow-up on participants with TB/DM including those who have died. The strength of the study lies in the triangulation of data obtained through interviews and literature showing similar trends with our findings. Furthermore, the participants represented the country as a whole, coming from a diverse range of rural, semi-urban and urban areas.

## Conclusion

Individuals with dual TB/DM comorbidity are jeopardized by the current disjunction and disruption in healthcare practice, which jeopardizes their well-being and may have a negative impact on their outcome. As a result, an integrated approach to TB and DM care is required to avoid the numerous challenges associated with isolated TB and DM services. The implementation of an integrated approach can be aided by the establishment of task-shifting policies and the design of health management information system.

## Supplementary Material

Supplemental Material

## Paper context

The lack of clinical integration in individuals’ pathway to dual TB/DM therapy has proven a struggle. There is limited data on individuals’ treatment plans, obstacles, or how they think these services can be improved. Due to the disruption in the healthcare system, individuals with dual TB/DM found it difficult to receive services at various levels of healthcare facilities according to this study. For optimal clinical management of dual TB/DM, integrated approaches are required.
