# Supplementary material for: Experiences and perceptions of participants on the pathway towards clinical management of dual tuberculosis and diabetes mellitus in Tanzania
Source: Glob Health Action. 2022 Nov 28;15(1):2143044. doi: 10.1080/16549716.2022.2143044 (PMC9894537; doi:10.1080/16549716.2022.2143044)
Supplement: Supplemental Material [file ZGHA_A_2143044_SM9788.docx]

**Appendix**

**Thematic Interview guide – English and Swahili.**

1. **Respect for patients’ values, preferences and expressed needs**

- Please tell me, what was your experience when you reached this health facility? (Clinic or wards).
- Tafadhali niambie uzoefu wako ulipofika katika kituo hiki cha afya? (Kliniki au wodini).

*Probe:*

1. *Can you explain how attentive are the health care providers towards your treatment?*

*Ni kwa jinsi gani watoa huduma ya afya wanakuwa makini wanapo kupa huduma.*

1. *Can you please tell me your views on the respect given to you by the health care providers in this facility?*

*Je, unaweza kunieleza maoni yako juu ya heshima uliyopewa na watoa huduma katika kituo hiki cha afya?*

- What other problems you were able to address to the health care providers apart from what brought you to the health facility?
- Unaweza kuelezea matatizo mengine uliyoweza kuwaeleza watoa huduma tofauti na lile lililo kuleta hospitali?

1. **Accessibility of care**

- Can you explain how you receive services in this health facility?
- Unaweza kuelezea namna unavyopata huduma katika kituo hiki cha afya?

*Probe:*

1. *How accessible are the services? / Je upatikanaji wa huduma ukoje?*
2. *How are the services provided?/Je huduma zinazotolewaje?*
3. *How many times do you come for treatment?/ Unalazimika kuja mara ngapi kupata huduma?*
4. **Coordination and integration of care**

- What is your opinion about the arrangement and integration of care?
- Je, nini maoni yako juu ya huduma inayotolewa hapa hospitalini kuhusu magonjwa yako?

*Probe:*

1. *What is your view in regards to integrated care?/ Nini maoni yako juu ya huduma ya pamoja?*
2. *What challenges arise with this arrangement and integration of care?*

*Ni changamoto gani unaziona katika utoaji wa huduma hii ya pamoja?*

1. **Emotional support and alleviation of fear and anxiety**

- Can you please tell me your feelings when you first heard that you are suffering from TB/DM
- Tafadhali nielezee hisia zako ulipogundua kwa mara ya kwanza una ugonjwa ya kifua kikuu na kisukari

*Probe:*

1. *Explain your feelings towards your illness? (TB/DM)*

*Elezea hisia zako juu ya magonjwa uliyonayo?*

- Can you tell me how the health care providers supported you in this situation?
- Je, unaweza kuniambia ni kwa jinsi gani watoa huduma ya afya walikusaidia katika hali hii?

*Probe:*

1. *How did they notice/detect your feelings?*

*Ni kwa jinsi gani walitambua hisia zako?*

1. *How were your feelings addressed?*

*Ni kwa jinsi gani hisia zako zilishughulikiwa?*

1. **Patient relationship and partnership in care**

- Can you please explain the way you see your relationship with the health care providers?
- Tafadhali niambie unavyoona mahusiano yako na watoa huduma ya afya?
- Explain to me how you have been involved or engaged by the health care providers in your treatment process?
- Nieleze ni kwa jinsi gani umeshirikishwa na watoa huduma ya afya juu ya matibabu ya ugonjwa wako?

*Probe:*

1. *In which ways were you given the opportunity to contribute to your treatment plan?*

*Ni kwa namna gani ulipewa fursa ya kuchangia katika matibabu yako?*

1. *How was your opinion taken by the health care provider?*

*Maoni yako yalichukuliwaje na watoa huduma?*

1. **Information and health promotion**

- What information did you get about your illness from the health care providers?
- Ulipata taarifa gani juu ya ugonjwa wako (TB/DM) kutoka kwa watoa huduma ya afya?

*Probe:*

1. *What are the causes of your disease? / Ugonjwa wako unasababishwa na nini?*
2. *What are the preventive measures of TB/DM?*

*Je, ni kwa jinsi gani unaweza kuzuia au kudhibiti huu ugonjwa wa TB/DM?*

1. **Involvement of family and friends**

- Can you explain your opinion in involving a family member or a friend in your treatment plan?
- Unaweza kuelezea maoni yako juu ya kuhusisha wanafamilia au rafiki katika mpango wa matibabu yako.
- After receiving your diagnosis, is there anybody else who was involved by the health providers about your diagnosis?
- Baada ya kufahamu ugonjwa wako, je, kuna mtu mwingine aliyehusishwa juu ya afya yako na mtoa huduma ya afya?

If Yes: Who are these? And why? Kama Ndio: Ni wakina nani? Na Kwanini?

If No: Why? Kama Hapana: Ni kwanini?

1. **Continuity and transition of care**

- How do the health care providers ensure continuity and transition of care?
- Je, ni kwa namna gani watoa huduma ya afya wanahakikisha matibabu yako ni endelevu?

*Probe:*

1. *How is the arrangement of the next appointment?*

*Je, kuna mpango gani uliowekwa kuhusu ufwatiaji wa matibabu yako?*

1. *Which other services do you receive, apart from what you are currently receiving?*

*Huduma gani zingine unapata kando ya hizi unazopatiwa?*

1. **Patient satisfaction**

- Kindly explain to what extent have the services met your needs and expectations?
- Tafadhali nieleze ni kwa namna gani huduma inayotolewa imekidhi mahitaji yako na matarajio yako ya kiafya?

*Probe: In regards to: / Kuhusiana na:*

1. *Time attended by the health provider / Muda unaonwa na mtoa huduma ya afya*
2. *Waiting time / Muda wa kusubiri matibabu*
3. *Availability of the Prescribed medications / Upatikanaji wa dawa unazo andikiwa*
4. *Communication with staff members / Mawasiliano au lugha inayotumiwa na watoa huduma ya afya.*
5. *Cost for health care services / Gharama za matibabu*
6. *Education given / Elimu itolewayo na watoa huduma ya afya juu ya afya yako*

THE END/MWISHO
